# A Retrospective Chart Review: The Prevalence of Hyponatremia Among Elderly Inpatients in a Tertiary Care Centre in Saudi Arabia

**DOI:** 10.7759/cureus.22960

**Published:** 2022-03-08

**Authors:** Nisreen Jastaniah, Renad A Sagim, Rehan M Sanyour, Duaa M Alamri, Rasha H Bajandouh, Esraa A Shaheen, Alaa Althubaiti

**Affiliations:** 1 Medicine, King Saud Bin Abdulaziz University for Health Sciences College of Medicine, Jeddah, SAU; 2 Research, King Abdullah International Medical Research Center, Jeddah, SAU

**Keywords:** comorbidity, medication, elderly, prevalence, hyponatremia

## Abstract

Introduction: Hyponatremia is commonly seen in inpatient care; however, the availability of data regarding the prevalence of hyponatremia in the elderly in Jeddah, Saudi Arabia is insufficient.

Objectives: The objective of this study is to estimate the prevalence of hyponatremia in a sample of older inpatients.

Materials and Methods: A retrospective chart review conducted in a tertiary care hospital, Jeddah, Saudi Arabia included all patients 60 years and older who were admitted to the internal medicine department between January 2017 and November 2020. Patients with hyponatremia were identified by looking through the hospital’s laboratory database of inpatients. Patients’ demographics, serum sodium level, medication history, and disease history were recorded and those with pseudohyponatremia were excluded.

Results: During the study period, 322 out of a total of 2,893 elderly patients admitted to the internal medicine department were diagnosed with hyponatremia (11.1%). Of these patients, 183 (56.8%) were female and 139 (43.2%) were male. Hyponatremia was more prevalent in patients with cardiovascular diseases (84.2%), followed by endocrine diseases (73.3%). The most prescribed medications for the studied hyponatremic patients were proton pump inhibitors (38.8%), diuretics (29.8%), angiotensin-converting enzyme inhibitors/angiotensin receptor blockers (22.4%), all of which are commonly known to be associated with hyponatremia.

Conclusions: Hyponatremia has severe consequences if left untreated. Therefore, estimating the prevalence of this condition in the older population will direct more clinical attention to evaluate the serum sodium level on a regular basis. It is recommended that future studies focus on finding an association between hyponatremia and older patients with multi-drug usage as well as identifying the causes of hyponatremia.

## Introduction

Electrolytes have an essential function in the human body’s balance. Sodium ion is one of the essential electrolytes that plays a significant role in various body functions by maintaining water balance, providing proper transmission of nerve signals and regulating muscle function. Thus, imbalances in sodium levels can result in health-related problems which range from nausea and malaise to lethargy, altered level of consciousness, seizures, headache, and coma if the condition is severe. The normal range of sodium level in the blood is between 135 and 145 millimoles per liter (mmol/L). Disturbances in the sodium level can cause significant disorders such as hyponatremia, which is a condition of a low serum sodium concentration (less than 135 mmol/L) [1-3]. Hyponatremia develops mainly from solute dilution, solute depletion, or a combination of both. Solute dilution results from overconsumption of water or the failure of the kidneys to excrete water, whereas solute depletion results from low salt intake or increased excretion of sodium by the kidneys [4]. Hyponatremia usually develops as a complication of some conditions, including cardiac, renal, endocrine, neurological and respiratory diseases; it can also result from the use of some medications, such as diuretics or antidepressants [5]. Although hyponatremia is widespread, the elderly are at higher risk of developing this disorder because they are more prone to having conditions or using medications that lead to the development of hyponatremia [6]. Furthermore, the fact that the majority of hyponatremic patients present with nonspecific symptoms, such as nausea and headache, or without symptoms, makes the disorder difficult to be diagnosed with a physical assessment only [7]. Another challenge for diagnosing hyponatremia is that its symptoms depend on the degree of severity of hyponatremia, which can range from mild (130-134 mmol/L) to moderate (125-129 mmol/L) to severe (less than 125 mmol/L). When correcting hyponatremia, inpatient care teams should take into consideration that restoring sodium levels too rapidly raises the risk of neurological conditions, such as osmotic demyelination syndrome (ODS), which is a type of brain injury [3].

Globally, the prevalence of hyponatremia among patients 18 years old and above varies depending on the studied population. For example, it is estimated to range from 1.72% in a sample from the community of the United States (US), to 17.5% and 32.5% in hospitalized patients of China and Switzerland, respectively. The association between hyponatremia and patient characteristics such as age and gender has been investigated thoroughly. The results have shown that the prevalence of hyponatremia increases with age [8-10]. However, there are conflicting results regarding the association between gender and the prevalence of hyponatremia. For instance, unlike the Swiss study, hyponatremia was higher in females in the US study [8,9].

In a study conducted over four years starting in 2013 on elderly patients in China, the overall prevalence of hyponatremia among older hospitalized patients was 24.7%. In addition, the study categorized the hyponatremic patients according to their primary diseases, and the results showed that patients with respiratory diseases accounted for 25% of patients, cardiovascular diseases for 19.9%, kidney disease for 3.9%, and endocrine diseases for 1.0% of patients. The study also categorized the patients according to their clinical use of drugs, including loop diuretics (57.4%), potassium-preserving diuretics (29.5%) and thiazide diuretics (12.5%) [5]. In a study conducted in Riyadh, Saudi Arabia, the overall prevalence of hyponatremia in inpatients taking indapamide (a thiazide-like diuretic) was 37.3%, whereas it was 38.7% in patients using hydrochlorothiazide (thiazide diuretic). However, the prevalence of hyponatremia was estimated based on a single risk factor rather than on a multifactorial overview [11].

The available research on hyponatremia in Saudi Arabia has not focused on the prevalence of hyponatremia in the elderly, which is the focus of this research. For instance, one study focused on the pediatric age group, which differs physiologically from the elderly in total body water, which has strong implications in the development of hyponatremia [12]. Although this issue is commonly seen in clinical practice, the availability of data on the prevalence of hyponatremia in the elderly in Jeddah, Saudi Arabia is lacking.

Because hyponatremia in the geriatric population is a common electrolyte disorder in clinical practice [6], a study of this condition will help to raise awareness and draw clinicians’ attention to patients who are at higher risk of developing hyponatremia, and thus, serious complications which might lead to morbidity and mortality can be monitored and limited. The aim of this study is to estimate the prevalence of hyponatremia in elderly patients between January 2017 and November 2020 and to determine the most common medication type and comorbidities among these patients.

## Materials and methods

Study settings and subjects 

This was a retrospective chart review conducted in a medical city in Jeddah, Saudi Arabia, which is a tertiary care center established in July 1982 with a 751-bed capacity. The participants were selected from the internal medicine department which includes dermatology, endocrinology, gastroenterology, geriatric medicine, infectious diseases, internal medicine, nephrology, neurology, physical medicine and rehabilitation, pulmonology, and rheumatology. This department’s services include diagnosis, management, and prevention of diseases. This study included males and females, and Saudi and non-Saudi patients aged 60 and older between January 2017 and November 2020. All patients with hyponatremia (serum sodium less than 135 mmol/L) were identified by looking through the hospital’s laboratory database of inpatients. All patients’ serum sodium levels were analyzed, and those with hyperglycemia or hypertriglyceridemia had their serum sodium values corrected to exclude patients with pseudohyponatremia [13].

Data collection 

The data collection tool recorded patients’ demographic variables (age and sex), serum sodium level (mild 130-134 mmol/L, moderate 125-129 mmol/L, or severe <125 mmol/L), well known medications that induced hyponatremia including (diuretics, proton-pump inhibitors [PPIs], selective serotonin reuptake inhibitors [SSRIs], non-steroidal anti-inflammatory drugs [NSAIDs], angiotensin-converting enzyme inhibitors [ACEIs]/angiotensin receptor blockers [ARBs] and antipsychotics) [5]. In addition, system-related diseases, and malignancy were recorded. The system-related diseases were categorized into ten classifications: respiratory, cardiovascular, renal, endocrine, hepatobiliary, neurological, genitourinary, gastrointestinal, orthopedics, and other lists (include dermatological disorders). The body mass index (BMI) of the patients was not recorded when hyponatremia was diagnosed and, therefore, was not reported in this study.

Data analysis 

Normality assumption was examined using the Kolmogorov-Smirnov test. Qualitative variables are presented as frequency and percentage, while quantitative variables are presented as mean (standard deviation) or median, minimum, and maximum. A 95% confidence interval (CI) for the prevalence of severity of hyponatremia was estimated. The Kruskal-Wallis test was used to compare the age at diagnosis among the severity groups of hyponatremia. The Chi-square was used to compare categorical data. For cases where patients had more than one comorbidity and using more than one type of medication, Cochran’s Q test was used [14]. A P-value <0.05 was considered statistically significant. JMP software (John’s Macintosh Project) and Microsoft Office Excel were used for data entry and analysis.

## Results

Prevalence of hyponatremia

The overall mean level of sodium was 122.9 (6.27) mmol/L. Of a total of 2,893 elderly patients admitted to the internal medicine department during the study period, 322 were diagnosed with hyponatremia. The estimated prevalence of hyponatremia was 11.1% (95% CI, 9.9-12.3%). Among the hyponatremic patients, 17% were diagnosed with mild hyponatremia, 22.4% were diagnosed with moderate hyponatremia and 60.6% were diagnosed with severe hyponatremia. Table 1 presents the prevalence of hyponatremia among different degrees of severity.

**Table 1:** Prevalence of hyponatremia in different degrees of severity. CI: confidence interval

	Mild hyponatremia	Moderate hyponatremia	Severe hyponatremia
Frequency	55	72	195
Prevalence (95%CI)	17(13.4–21.6)	22.4(18.2–27.2)	60.6(55.1–65.7)

Patients’ characteristics 

The median age of the studied sample was 75 years (range: 60-109). Of the 322 patients, 183 (56.8%) were female and 139 (43.2%) were male. No significant associations were found between severity of hyponatremia, age at diagnosis, and sex (p = 0.39 and 0.58, respectively) (Table 2). In total 316 (98.1%) patients had documented comorbidities and 196 (60.9%) were on medications. No significant associations were found between severity of hyponatremia, presence of comorbidities, and medication status (p=0.94 and 0.64, respectively). 

**Table 2:** Patient characteristics based on the severity of hyponatremia

Characteristics	Total (n=322)	Mild hyponatremia (n=55)	Moderate hyponatremia (n= 72)	Severe hyponatremia (n= 195)	p-value
Age at diagnosis, median (minimum-maximum) years	75(60-109)	73(60-95)	75(60-109)	75(60-96)	0.39
Sex					
Female	183(56.8)	28(15.3)	43(23.5)	112(61.2)	0.58
Male	139(43.2)	27(19.4)	29(20.9)	83(59.7)	
Comorbidities					
Yes	316(98.1)	54(17.1)	71(22.5)	191 (60.4)	0.94
No	6 (1.9)	1(16.7)	1(16.7)	4(66.7)	
Medication					
Yes	196(60.9)	34 (17.3)	47 (24)	115(58.7)	0.64
No	126(39.1)	21(16.7)	25(19.8)	80(63.5)	

Types of comorbidities and medications

**Figure 1:**
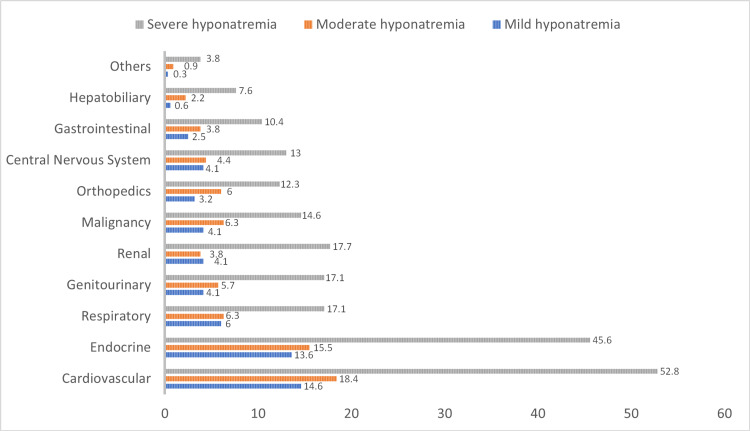
Distribution of various comorbidities in the hyponatremic patients. Percentages of total of 316 patients with comorbidities are presented. The category others refers to integumentary and lymphatic systems.

**Figure 2:**
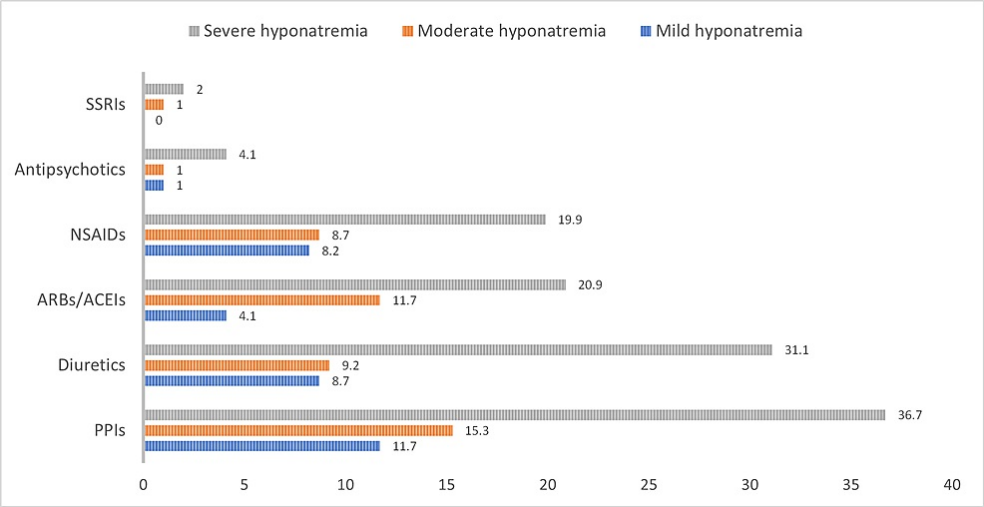
Distribution of medications in each group of hyponatremic patients (a total of 196 valid cases). PPIs: proton-pump inhibitors, ACEIs/ARBs: angiotensin converting enzyme inhibitors/angiotensin II receptor blockers, NSAIDs: nonsteroidal anti-inflammatory drugs, SSRIs: selective serotonin reuptake inhibitors

Figure 1 presents the percentages on the basis of observed patients with comorbidities (i.e. cases, a total of 316 observations, six patients had no documented comorbidities). It is evident from Figure 1 that cardiovascular diseases are the most common type of comorbidity among the hyponatremic patients, where 271 of the patients had a cardiovascular disease (85.8%), followed by endocrine diseases (n=236, 74.7%), respiratory diseases (n=93, 29.4%), and genitourinary diseases (n=85, 26.9%). Figure 2 presents the percentages on the basis of observed medications (i.e. a total of 196 valid cases). The most frequently used drugs were PPIs (n = 125, 63.8%), diuretics (n = 96, 49%), ACEIs/ARBs (n = 72, 36.7%), and NSAIDs (n = 72, 36.7%). In addition, SSRIs and antipsychotics were the least used medications (3.1% and 6.1%, respectively). Thus, the mean number of medications is approximately 2 in this study. Detailed description of the frequencies and row percentages based on each comorbidity and medication type across the hyponatremic groups are provided in Table 3 and Table 4. No significant association between comorbidities and medication types and the severity of hyponatremia was found.

**Table 3:** Distribution of comorbidities across hyponatremic patients.

Comorbidities	Total	Mild hyponatremia	Moderate hyponatremia	Severe hyponatremia	P value
Cardiovascular	271 (85.8)	46 (17.0)	58 (21.4)	167 (61.6)	
Endocrine	236 (74.7)	43 (18.2)	49 (20.8)	144 (61.0)	
Respiratory	93 (29.4)	19 (20.4)	20 (21.5)	54 (58.1)	
Genitourinary	85 (26.4)	13 (15.3)	18 (21.2)	54 (63.5)	
Renal	81 (25.6)	13 (16.1)	12 (14.8)	56 (69.1)	
Malignancy	79 (24.5)	13 (16.5)	20 (25.3)	46 (58.2)	0.75
Orthopedics	68 (21.5)	10 (14.7)	19 (27.9)	39 (57.4)	
Central Nervous System	68 (21.5)	13 (19.1)	14 (20.6)	41 (60.3)	
Gastrointestinal	53 (16.8)	8 (15.1)	12 (22.6)	33 (62.3)	
Hepatobiliary	33 (10.4)	2 (6.06)	7 (21.2)	24 (72.7)	
Others	16 (5.1)	1 (6.25)	3 (18.8)	12 (75.0)	
Total of 316 patients with one or more comorbidities. N(%) are reported.					

**Table 4:** Distribution of medications in each group of hyponatremic patients. PPIs: proton-pump inhibitors, ACEIs/ARBs: angiotensin converting enzyme inhibitors/angiotensin II receptor blockers, NSAIDs: nonsteroidal anti-inflammatory drugs, SSRIs: selective serotonin reuptake inhibitors

Medication type	Total	Mild hyponatremia	Moderate hyponatremia	Severe hyponatremia	P value
PPIs	125 (63.8)	23 (18.4)	30 (24)	72 (57.6)	
Diuretics	96 (49)	17 (17.7)	18 (18.8)	61 (63.5)	
ARBs/ACEIs	72 (36.7)	8 (11.1)	23 (31.9)	41 (56.9)	0.40
NSAIDs	72 (36.7)	16 (22.2)	17 (23.6)	39 (54.2)	
Antipsychotics	12 (6.1)	2 (17.8)	2 (16.7)	8 (66.7)	
SSRIs	6 (3.1)	0 (0.0)	2 (33.3)	4 (66.7)	
Total of 196 patients receiving medication. N(%) are reported.					

## Discussion

Hyponatremia is one of the electrolyte disorders that is common in elderly patients. If it is not treated promptly, serious consequences may occur. The study aimed to estimate the prevalence of hyponatremia in elderly patients admitted to the internal medicine department in a tertiary care center in Jeddah. A previous study in Italy on a community-based follow-up study by general practitioners reported that the prevalence of hyponatremia was 8% in patients aged 65 or older [15]. Another study in an internal medicine department in the southeast of China estimated the prevalence of hyponatremia to be 3.37% in patients aged older than 18, however, it is unknown whether hyponatremic patients are elderly [16]. Our results showed the prevalence of hyponatremia in the inpatients of internal medicine department aged 60 and older to be 11.1%.

Commonly prescribed drugs for elderly patients can exacerbate preexisting hyponatremia or induce iatrogenic hyponatremia by different mechanisms. For example, volume clearance, the impact of sodium ion re-uptake in renal tubules, and the abnormal secretion of anti-diuretic hormone are mechanisms associated with drug-induced hyponatremia [5,17]. Previous studies have shown that the most used drugs among older hyponatremic patients are potassium-sparing diuretics, loop diuretics, thiazide diuretics, PPIs, ACEIs/ARBs and NSAIDs [5]. Another study demonstrated that diuretics are known to be associated with hyponatremia, especially in the outpatient geriatric population. It has been reported that 28.8% of hyponatremic patients were taking diuretics [18]. Another study found that the use of ACEIs/ARBs was significantly more frequent in patients with severe hyponatremia than in patients with mild hyponatremia, which was similar to our findings [5].

Hyponatremia can develop as a result of various conditions [18]. In our study, cardiovascular and endocrine disorders were the comorbidities found most frequently in patients with hyponatremia. A well-known mechanism of cardiovascular diseases has been attributed to an impairment in the excretion of diluted urine, which disturbs water balance and leads to an increase in extracellular fluid, causing hyponatremia [19]. Furthermore, diabetes mellitus, as an endocrine disorder, causes an increased glucose glomerular filtration, which leads to an increase in fluid excretion by osmotic diuresis, resulting in hypovolemia and hyponatremia [20]. Previous studies have found that hypertension followed by diabetes mellitus were the most prevalent comorbidities in elderly hyponatremic patients [17,21].

This research has several strengths. To the authors' knowledge, It is the first study conducted in Saudi Arabia to estimate the prevalence of hyponatremia in a geriatric population. The studied population was unselected and included all hyponatremic patients admitted to the internal medicine department during the study period. Furthermore, the serum sodium level was corrected for patients with hypertriglyceridemia and hyperglycemia; thus, patients with pseudohyponatremia were excluded. This study also has some limitations. First, it was based on data from a single center and it included the elderly inpatients in the internal medicine department only. Second, there was no follow-up period as patients’ serum sodium level was measured once on admission. Third, subtypes of hyponatremia (euvolemic, hyper/hypo-volemic) were not discussed.

We recommend that future research investigate the association between hyponatremia and the elderly with multi-drug usage to understand its effect on decreasing patients’ serum sodium levels as well as identify the causes of hyponatremia to help reduce its incidence. 

## Conclusions

Hyponatremia is repeatedly observed in the elderly and was seen in 11.1% of the patients included in this study. Comorbidities frequently occur with hyponatremia, with cardiovascular disorders being the most prevalent. In addition, PPI medications were among the medications most frequently used by the hyponatremic patients in our study. Estimating the prevalence of hyponatremia in the geriatric population will increase clinicians’ awareness of the need to regularly monitor and manage serum sodium levels in elderly patients, thus preventing and reducing its serious complications.
